# Artificial Intelligence and Cardiovascular Genetics

**DOI:** 10.3390/life12020279

**Published:** 2022-02-14

**Authors:** Chayakrit Krittanawong, Kipp W. Johnson, Edward Choi, Scott Kaplin, Eric Venner, Mullai Murugan, Zhen Wang, Benjamin S. Glicksberg, Christopher I. Amos, Michael C. Schatz, W. H. Wilson Tang

**Affiliations:** 1 Section of Cardiology, Baylor College of Medicine, Houston, TX 77030, USA; 2 Department of Cardiovascular Medicine, NYU Langone, New York, NY 10016, USA; scott.kaplin@nyulangone.org; 3 The Hasso Plattner Institute for Digital Health at the Mount Sinai, Icahn School of Medicine at Mount Sinai, New York, NY 10029, USA; kipp.johnson@icahn.mssm.edu (K.W.J.); benjamin.glicksberg@mssm.edu (B.S.G.); 4 Human Genome Sequencing Center, Department of Molecular and Human Genetics, Baylor College of Medicine, Houston, TX 77030, USA; venner@bcm.edu; 5 Department of Genetics and Genomic Sciences, Institute for Next Generation Healthcare, Icahn School of Medicine at Mount Sinai, New York, NY 10029, USA; 6 Google Health Research, Google, Mountain View, CA 94043, USA; edwardchoi@kaist.ac.kr; 7 Human Genome Sequencing Center, Department of Software Development, Baylor College of Medicine, Houston, TX 77030, USA; mullai.murugan@bcm.edu; 8 Robert D. and Patricia E. Kern Center for the Science of Health Care Delivery, Mayo Clinic, Rochester, MN 55905, USA; wang.zhen@mayo.edu; 9 Division of Health Care Policy and Research, Department of Health Sciences Research, Mayo Clinic, Rochester, MN 55905, USA; 10 Dan L Duncan Comprehensive Cancer Center, Baylor College of Medicine, Houston, TX 77030, USA; chris.amos@bcm.edu; 11 Department of Computer Science, Johns Hopkins University, Baltimore, MD 21218, USA; mschatz@cs.jhu.edu; 12 Department of Biology, Johns Hopkins University, Baltimore, MD 21218, USA; 13 Department of Cardiovascular Medicine, Heart and Vascular Institute, Cleveland Clinic, Cleveland, OH 44195, USA; tangw@ccf.org; 14 Department of Cellular and Molecular Medicine, Lerner Research Institute, Cleveland, OH 44195, USA; 15 Center for Clinical Genomics, Cleveland Clinic, Cleveland, OH 44195, USA

**Keywords:** genomics, AI, genetics, deep learning, cardiovascular disease, cardiology, machine learning, artificial intelligence

## Abstract

Polygenic diseases, which are genetic disorders caused by the combined action of multiple genes, pose unique and significant challenges for the diagnosis and management of affected patients. A major goal of cardiovascular medicine has been to understand how genetic variation leads to the clinical heterogeneity seen in polygenic cardiovascular diseases (CVDs). Recent advances and emerging technologies in artificial intelligence (AI), coupled with the ever-increasing availability of next generation sequencing (NGS) technologies, now provide researchers with unprecedented possibilities for dynamic and complex biological genomic analyses. Combining these technologies may lead to a deeper understanding of heterogeneous polygenic CVDs, better prognostic guidance, and, ultimately, greater personalized medicine. Advances will likely be achieved through increasingly frequent and robust genomic characterization of patients, as well the integration of genomic data with other clinical data, such as cardiac imaging, coronary angiography, and clinical biomarkers. This review discusses the current opportunities and limitations of genomics; provides a brief overview of AI; and identifies the current applications, limitations, and future directions of AI in genomics.

## 1. Introduction

Multiple diseases of the cardiovascular system are associated with genetic polymorphisms including both common conditions, such as hypercholesterolemia [1,2] and less common conditions, such as cardiac channelopathies [3], cardiomyopathies [4], aortopathies [5], and various structural and congenital diseases of the heart and great vessels [6]. Given that the fields of cardiovascular genetics and precision medicine are rapidly evolving, it is unsurprising that recently published guidelines include an increased focus on genetic testing. The 2020 Scientific Statement From the American Heart Association (AHA) on Genetic Testing for Inherited Cardiovascular Diseases recommended testing specific genes in certain monogenic cardiovascular diseases (CVDs) in appropriate clinical circumstances [7] (e.g., *LDLR*, *APOB*, and *PCSK9* genes for familial hypercholesterolemia, and *TTN*, *LMNA*, *MYH7*, *TNNT2*, *BAG3*, *RBM20*, *TNNC1*, *TNNI3*, *TPM1*, *SCN5A*, and *PLN* genes for dilated cardiomyopathy). The 2021 Scientific Statement from the AHA on Genetic Testing for Heritable Cardiovascular Diseases in Pediatric Patients also recommended cardiovascular genetic testing in children as an important component in determining the risk of developing heritable cardiovascular diseases in adulthood [8]. With advancements in technology, several recent genetic studies have revealed potential targets for CVD screening and therapies. For example, a recent genome-wide association study of 2780 cases and 47,486 controls identified 12 genome-wide susceptibility loci which were significant for hypertrophic cardiomyopathy (HCM), and found that single-nucleotide polymorphism heritability indicated a strong polygenic influence, especially for sarcomere-negative HCM (64% of cases; h2g = 0.34 ± 0.02) [9]. Another recent study of patients with hereditary transthyretin (TTR) cardiac amyloidosis with polyneuropathy showed that administration of NTLA-2001 led to a decrease in serum TTR protein concentrations through targeted knockout of *TTR*. Hence, genetic screening of TTR may, thus, prove to be increasingly useful in the future as it may allow susceptible patients to be identified and treated appropriately at an earlier stage of disease [10]. On the other hand, genetic testing in polygenic CVDs, with their inherently more complicated genetic etiology, remains challenging. 

Artificial intelligence (AI) is a discipline of computer science that aims to mimic human thought processes, learning capacity, and knowledge storage [11]. A central tenet of AI is learning the value of potential choices rather than rigidly following predetermined thresholds or procedures, e.g., optimizing the selection of variants to maximize the predictive accuracy for disease risk rather than using a predetermined list. AI involves several components, including machine learning and deep learning, with increasing potential to explore novel CVD genotypes and phenotypes, among many other exciting opportunities. In this review, we summarize several important current limitations of genomics; provide a brief overview of AI; and identify the current applications, limitations, and potential future directions of AI in cardiovascular genetics.

## 2. Genetic Testing Gap in Cardiovascular Diseases

The majority of CVDs and cardiovascular risk factors have a significant genetic component, which is most commonly polygenic in origin [1,2]. Current clinical practice utilizes a patient’s medical history, family history, physical examination, cardiac biomarkers, and various modalities of cardiac imaging to establish diagnoses and to stratify risks. Despite rapid advances and availability of genetic testing panels, clinicians seldom utilize genetic testing as part of their initial patient assessments beyond cases with a known family history of genetic, inherited CVDs (e.g., HCM, arrhythmogenic right ventricular cardiomyopathy (ARVC), long QT syndrome (LQTS), or catecholaminergic polymorphic ventricular tachycardia (CPVT)). This lack of routine testing as part of care pathway creates a “diagnostic gap” (i.e., a delay in time from disease manifestation to establishing a definitive diagnosis) that can lead to inappropriate or ineffective treatment in patients suffering from inherited CVDs. In a recent study from Baylor College of Medicine’s Human Genome Sequencing Center, 84% of surveyed physicians reported medical management changes, including specialist referrals, cardiac testing, and medication changes, after receiving the results of a panel of genes associated with CVDs [12]. 

Despite its demonstrated clinical relevance, current guidelines only recommend genomic testing for a small number of cardiac conditions (e.g., HCM, familial hypercholesterolemia), limited by the relatively few genetic tests that are currently available and the lack of strong studies in cardiovascular genetics [13,14]. For example, Brugada syndrome has a large number of potentially pathogenic genetic variants (e.g., *CACNA1C*, *GPD1L*, *HEY2*, *PKP2*, *RANGRF*, *SCN10A*, *SCN1B*, *SCN2B*, *SCN3B*, *SLMAP*, and *TRPM4*) but current guidelines continue to recommend a comprehensive genetic analysis for only Brugada syndrome caused by the *SCN5A* genetic variant [15,16]. With advancements in genetic testing technologies, preemptive genetic testing for various cardiomyopathies may be useful in the presence of an asymptomatic type 1 Brugada ECG pattern, family history of dilated cardiomyopathy, or the development of spontaneous coronary artery dissection (SCAD). While a recent study by Murdock and colleagues demonstrated the diagnostic potential of genetics guided coronary artery disease (CAD) risk factor management based on *LPA* polymorphisms and polygenic risk, genetic testing for a selection of well-understood variant–phenotype associations remains very limited (i.e., a “treatment gap”) [12]. With further research and development, comprehensive genetic testing could become routinely used in clinical cardiovascular practice and applied to primary disease prevention and the facilitation of precision cardiovascular medicine.

## 3. Next Generation Sequencing (NGS) in the Modern Clinic

Genomics is becoming nearly ubiquitous in biomedical research [17]. Large-scale sequencing efforts have revolutionized our understanding of the complex genetic interrelationships involved in the pathogenesis of most cardiovascular conditions [18]. The tremendous advancements in genomic research are largely driven by the advent of NGS, which has led to the discovery of novel associations and the ability to more easily assess genetic heterogeneity across patients. Several categories of NGS include: (1) whole genome sequencing (WGS); (2) whole exome sequencing (WES), where the sequencing is concentrated over the protein-coding regions of the genome (~2% of the genome); and (3) gene panels, where very deep coverage (>100× coverage) is generated for a select number of genes. Both WGS and WES allow for the accurate identification of single-nucleotide variants (SNVs), large copy number variations (CNVs), small insertion deletions (InDels), and information on variant frequencies in different populations [19]. Because WGS examines the noncoding regions of the genome, it offers a more comprehensive appraisal of both small and large genomic risk variants for CVDs. However, WGS is more costly and time-consuming than WES, and may be limited by lower depth [20,21]. Conversely, the results of WES, while more limited in scope, are typically viewed as more straightforward to interpret and historically have been a useful method to identify variants causing Mendelian disease. Panel-based NGS relies on high sequencing depth of previously determined important genetic loci, making this kind of testing more resource-efficient. However, the narrow focus of this type of assay results in decreased power to detect novel associations and is often less effective for assessing other types of genetic alterations, such as structural variants. Although NGS is now widely used due to its speed, robustness, and cost-effectiveness, orthogonal confirmation with the traditional Sanger sequencing method is sometimes still required for validation prior to clinical use [22,23,24].

Nonetheless, the implementation of AI to NGS and genomics has already been shown to accurately predict the consequences of genetic risk factors in CVDs [25,26], show the noncoding-variant effects in CVDs [27,28], find patients with cardiac amyloidosis [29,30], and initiate specific therapies from tumor sequencing [31] by integrating with electronic health records (EHRs) in several academic and medical institutions. Additionally, there are several direct-to-consumer genomics companies that use AI along with WGS and WES; however, to date, these applications have been limited by a lack of transparency in the algorithms they utilize due to their proprietary nature and commercial competition, as well as a lack of a consistent validation cohort, genomic guided clinical trials, and high-quality phenotype data that are consistently encoded and managed (Table 1). Although some direct-to-consumer companies have collaborated with academic institutions and published their methodologies, evidence for their clinical relevance remains scarce. 

## 4. Introduction of AI to Clinical Cardiovascular Genetics

**Figure 1:**
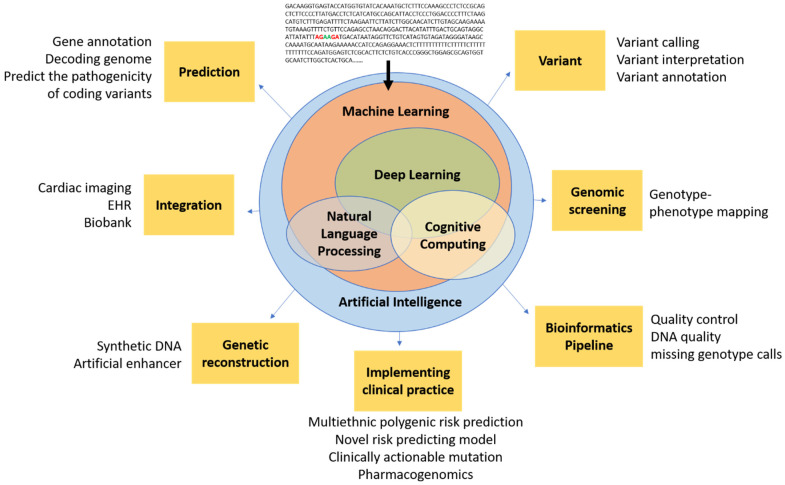
Conceptual schematic for artificial intelligence in cardiovascular genetics. Artificial intelligence encompasses a spectrum of concepts, including machine learning, NLP, and cognitive computing, which are generally enabled by deep learning and could ultimately be used in cardiovascular genomics for prediction, integration, reconstruction, bioinformatic techniques (e.g., pipeline, screening, variant analysis), and clinical practice. Artificial intelligence has the potential to filter raw genetic data into novel insights that could inform future clinical trials and, ultimately, clinical practice.

AI encompasses a broad range of applications for automated reasoning and inference, and is starting to have a major impact on clinical assessment and diagnosis. For example, in both United States of America (US) and United Kingdom (UK) datasets, AI outperformed human radiologists in screening mammography (greater than the AUC-ROC for the average radiologist by an absolute margin of 11.5%) and significantly reduced false positives and false negatives [32]. The most widely used groups of methods for pattern recognition in genomics include machine learning (ML) and deep learning (DL). Other AI approaches, for example natural language processing (NLP) and cognitive computing, are also starting to play a role in cardiovascular clinical care to enable more natural interactions between clinicians and computational systems [33,34,35]. Notably, the Food and Drug Administration (FDA) has been rapidly approving AI/ML-based medical devices and algorithms. Therefore, it is crucial for medical professionals to understand how best to utilize them. In a recent study using a web-based search for announcements of FDA approvals of AI/ML-based medical devices and algorithms, of the 64 found, 30 (46.9%), 16 (25.0%), and 10 (15.6%) were developed for the fields of radiology, cardiology, and internal medicine/general practice, respectively [36]. These AI approaches fundamentally work to train programs to recognize relationships within data. Table 2 provides examples of variant calling, reporting, and interpretation AI. Figure 1 demonstrates the potential of AI in cardiovascular genetics. 

### 4.1. Machine Learning and Deep Learning

Since it is origins in the 1940s, ML has used algorithmic and statistical techniques to process data for a variety of purposes and applications [64,65]. ML concepts, such as supervised machine learning (e.g., support vector machines to distinguish between cases and controls) and unsupervised machine learning (e.g., a variety of models to reduce highly dimensional data into lower dimensional space), are common tools in genome-wide association studies (GWAS). In contrast to these types of ML, DL is a time- and resource-intensive subtype of ML that can achieve higher performance via its ability to learn complex representations from the data, depending on the task. Recently, advancements in computational power have enabled the application of DL onto large data sets (i.e., “big data”) to build extremely expressive and complex multi-layer artificial neural networks (ANN) [66]. The initial success of DL began in image processing and recognition, where it can be used to recognize objects without explicitly defining the relevant features. For example, instead of trying to identify the specific contours of the nose, eyes, or mouth, the DL algorithm categorizes an object as a “face”, which is recognized through a more abstract representation automatically learned from prior training on a dataset. In CVD, DL has been applied to non-imaging data, improving the accuracy of patient risk stratification and relationship prediction in comparison to traditional models, such as the Framingham Risk Score; although, typically, DL outperforms other models only on non-tabular data where there are complex nonlinear features that can be learned in a highly connected model [67,68].

Both ML and DL have their advantages for clinical genetics and carry the potential to improve the capabilities of cardiovascular genetics. As mentioned above, ML and DL can be further classified into supervised [69] and unsupervised [70,71] approaches. In a supervised approach, a classifier learns to predict known outcomes (e.g., predict the effect of a *LAMP2* mutation and understand its relationship to the phenotype of Danon Disease), while an unsupervised approach learns to infer relationships within the dataset (e.g., to identify subsets of patients who may carry similar genetic features or disease risk factors). ML has also been applied for several different tasks in NGS [72]. Support vector machine (SVM) models (learning methods used for classification, regression, and outlier detection) are used in high dimension datasets, similar to those used for predicting polygenic risk factors for hypertension [73] or inherited arrhythmias [74]. More complex ANN models have been used to predict advanced coronary artery calcium through a large-scale GWAS [75] and inheritable dilated cardiomyopathy through SNVs [76]. These ML models can also be used to cluster low-expression genes in pulmonary arterial hypertension [77]. 

The complexity of DL architecture creates challenges when analyzing large genomic data. There are several steps to analyzing genomic data using DL. First, before performing DL analysis, genomic data must be transformed into an appropriate data set for analysis and the network architecture should be designed to solve the specific cardiovascular task. “One-hot encoding” is a vector-based approach that has emerged as the most common method to represent genomic sequences for DL analysis, although other numerical representations (e.g., vectors, matrices, or tensors) and image-based approaches (e.g., DeepVariant transforming BAM files to images) have been proposed [78]. The second step is to design the network architecture. The major components of network architecture design include the type and resolution of the input filters and layers, the depth and density of the network, and a decision on the loss function regularization strategy. Once the genomic data and network architecture parameters are set, training the network with back propagation can begin [79]. 

The next step is to train the network. During training, the model parameters are learned by the network from the training data provided relative to the labeled examples using backpropagation and other related gradient descent learning techniques. The major challenge of this task is collecting enough training data and optimizing the hyperparameters (e.g., initialization strategy, learning rate, regularization techniques) so that the network can learn a robust set of parameters for the given prediction. It may also be necessary to reconsider the overall network architecture if the performance remains low. Importantly, given enough training data, sufficient computational resources, and an appropriate network architecture, nearly any mathematical function can be learned, including highly abstract functions from genomics data or image data to a disease state. 

Once training is complete, the main task of prediction can begin (e.g., predicting gene function [80], pharmacogenomics outcome [19], or variant detection [81]) using supervised learning for genotype–phenotype mapping (e.g., SNV variations with phenotypes) or to apply the learned models (if the data are labeled) to novel datasets. This task is particularly challenging in cardiology because many cardiovascular conditions are heterogeneous and not well-defined. For instance, heart failure classification is largely based on ejection fraction (HFrEF, HFpEF, and HFmrEF) but ejection fraction assessment can be affected by angle-dependent and interoperability issues. Furthermore, current cardiovascular genetic datasets restrict access and contain a homogeneous population. The Million Veteran Program, one of the largest genetic and CVD datasets assembled, limits access to its data, and most other major public CVD genetic data sets are largely based on UK Biobank samples, which are from a largely Caucasian British population (94% Caucasian). 

Once training is complete, the creation and analysis of artificial nucleotide sequences, such as the creation of artificial human genomes [82] or artificial enhancers (“synthetic DNA”), using approaches such as generative adversarial networks (GANs), can be considered [83]. GANs are DL models that include two primary components: a generator and a discriminator. Generated DNA sequences are used as inputs for the discriminator to analyze if the model has generated a convincingly real biological sequence. This feedback is used to iteratively train the generator model to produce artificial sequences with increasingly realistic properties. For example, a recent study used a type of GAN (an auxiliary classifier generative adversarial network) to generate synthetic participants that closely resembled real participants from the SPRINT trial (Systolic Blood Pressure Trial) to facilitate exploratory analyses [84].

Using these techniques, DL has been successfully applied within genomics in several major projects, including DeepSEA (a DL-based sequence analyzer that can predict the epigenetic state in multiple cell types), and a subsequent DragoNN primer online training in academic institutions globally [27,85]. To date, convolutional neural networks (CNNs), recurrent neural networks (RNNs), autoencoders, and GANs have been the primary DL techniques used in genomics (Table 2). These approaches have been implemented for several tasks, including functional assessments of variants [28], AI-guided multiethnic polygenic risk score (PRS) generation [86]. and variant calling optimization [87]. Interestingly, the number of layers within DL architectures used in genomics has generally been far less than those used for image recognition, and, thus far, typically consist of only a few layers [27,79,88] with many hundreds to thousands of parameters [89]. 

**Figure 2:**
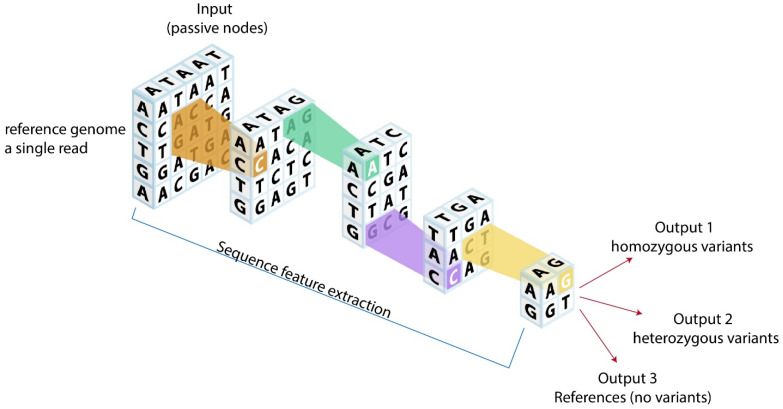
Potential analytic models for cardiovascular genomics. Reference genome or a single read could be fed into neural network models using convolutional genetic coding based on genetic structures. After neural network processing, outputs can be categorized into homozygous variants, heterozygous variants, and references (no variants), which could ultimately provide novel clinical genetic insights.

Given the broad variety of potential genomic data types (e.g., genetic variants, DNA methylation, gene expression, miRNA expression data, transcription factor binding, chromatin state, etc.), there is a growing trend to use DL to perform multi-faceted biological data integration. This strategy could be used to classify new CVD genotype–phenotype relationships, which could then result in the identification of novel therapeutic targets (e.g., new therapies based on genetic loci and left ventricular mass to volume ratio from cardiovascular magnetic resonance imaging, left ventricular end-diastolic pressure from echocardiography, or novel strain patterns from strain imaging) [25,90]. Using DL-guided WES in clinical practice to bridge the phenotype–genotype gap also shows promising utility [91]. DL could be used to reduce sequencing biases known to affect WES data analysis (e.g., coverage biases [92] or GC content bias [93,94]). Figure 2 demonstrates a typical DL model used in genomics. We have previously described several major DL libraries [65,66] and DL guidelines in cardiovascular medicine [66]. In addition, new open-source genomics libraries, such as Nucleus, which builds on top of TensorFlow, may be used for future DL in genomic research. At least one clinical trial (NCT03877614) is underway using DL in genetics and CVDs, including CAD, HFrEF, HCM, atrial fibrillation, pulmonary hypertension, and Fabry’s disease, compared to a healthy to low risk control group (atherosclerotic cardiovascular disease score <10%). In the future, DL could potentially be used to predict the future development of many CVDs using genomic findings as inputs.

### 4.2. Natural Language Processing

NLP is a set of computational methods that are able to understand language by analyzing its syntax and semantics. Major applications of NLP within medicine include analyzing progress notes [95], identifying critical illness [96], de-identifying patient records [97], reducing human workload of literature reviews [98], and predicting readmission from discharge summaries [99]. Within genomics, NLP has been used for gene recognition or normalization [100] and identifying gene–disease associations in heart failure [101]. Interestingly, NLP has also been used to predict genes for CAD [102,103], while other techniques rely on a combination of ML, DL, and NLP to predict gene alterations [63,64].

Advancements in NLP may incorporate clinical guidelines to automatically generate appropriate recommendations for CVD prevention in a patient’s discharge summary. For example, based on the current literature and the level I evidence available, NLP could recommend the most appropriate anticoagulation treatment for patients with a left ventricular thrombus. Another example would be NLP of admission notes to determine possible necessity for genetic screening. However, NLP must first understand the relevant clinical semantics (e.g., analyzing all literature in PubMed and clinical notes in EHRs) in order to provide appropriate clinical recommendations. Although ML algorithms are more often used for predictive analyses, ML algorithms are also able to perform NLP tasks using ML-based NLP models [104]. For example, the implementation of NLP-DL to review genes related to clinically actionable mutations is feasible [105]. Advanced AI techniques, such as deep reinforcement techniques, can be a powerful approach for NLP tasks for heterogeneous CVDs and genomics [106]. Deep reinforcement-based NLP models could, for example, potentially enhance traditional algorithms to identify mutations by working to rule out read errors. 

## 5. Current Limitations in Genomics and Potential Solutions with AI

**Figure 3:**
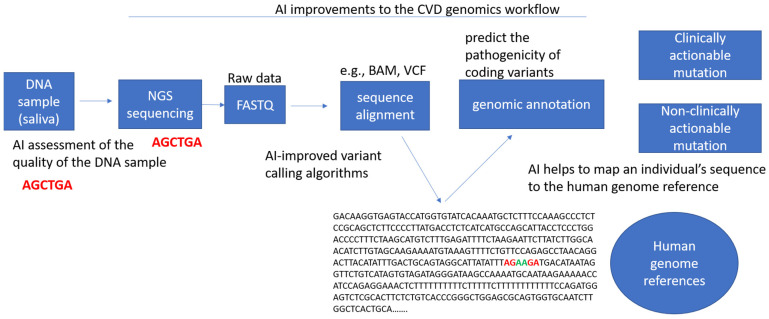
Potential artificial intelligence improvements to the workflow in cardiovascular genomics. This includes the assessment of the quality of genetic samples obtained (e.g., DNA, RNA, exome), the improvement of informatics pipelines for variant calling, the translation of clinical guidelines for variant interpretation, the transformation of genetic files (e.g., VCF to BAM, VCF to PED), the prediction of variant pathogenicity, the mapping of an individual’s sequence to genome references, and the identification of any clinically actionable mutations.

Below we describe the limitations in current genomic research and discuss how AI implementation can address these limitations and advance the field (Figure 3). 

### 5.1. Lack of Clinical and Technical Guidelines for Cardiovascular Genetics

Currently in clinical cardiovascular genetics, the guidelines do not specify which genes should be tested or how to validate the results. For example, the 2019 HRS Expert Consensus Statement on Evaluation, Risk Stratification, and Management of Arrhythmogenic Cardiomyopathy did not define how genetic testing should be validated or carried out in ARVC and other arrhythmogenic cardiomyopathies [107]. Similarly, the 2020 and 2021 scientific statements from the AHA on Genetic Testing for Heritable Cardiovascular Diseases in adult and pediatric patients did not specify how genetic testing should be validated or carried out in heritable cardiovascular diseases [7,8]. 

At a more rudimentary level, the Clinical Laboratory Improvement Amendment (CLIA) and the College of American Pathologists (CAP) have left many inconsistencies and regulatory gaps in their guidance for wet and dry labs [108], resulting in heterogeneous variant reporting. Moreover, CAP/CLIA regulations only require that validation is performed in the production environment, which may lead to unexpected errors in the production phase. Bioinformatics pipelines should be validated and tested for how precisely and sensitively variants are called in wet labs. Technical variability in the QC process, such as consistency of sequencing [109], QC standardization [110], and DNA quality [111,112], has been highly problematic; however, with current technologies, the accuracy of SNV is generally very robust (particularly if 30x or greater sequencing coverage is available). However, despite the advances in SNV analysis, structural variation calling continues to be highly variable and problematic. Automated QC systems using AI may decrease these issues by recognizing outliers and inconsistent data, identifying structural variations or small mutations from random errors and complex variants from long-read sequencing [113], and improving missing genotypes imputation [114]. While few studies have developed NLP-guided bioinformatics pipelines [115,116,117], ML-based pipelines have been more widely reported [118,119]. Unfortunately, most of these ML-based pipelines are not well validated across different databases, which may introduce population-specific biases. Given the variety of DL architectures (e.g., convolutional networks or encoders), DL models may be able to target and improve existing bioinformatics pipelines and variant classifications [120,121].

Another major barrier to current cardiovascular genetic research is the lack of professional recommendations for the clinical integration of genomics. Several clinical research projects using different genomics databases (e.g., UK Biobank [67], MESA [122], and ARIC [123]) have demonstrated accurate ML model discrimination and calibration (e.g., Brier score) for CVD risk prediction using genetics, but there are as yet no specific guidelines for genetic testing in clinical practice or regulatory guidance for direct-to-consumer products. This has also led to a lack of reimbursements for testing and a lack of incentives for routine testing. While most direct-to-consumer genetic testing companies are CAP/CLIA-certified, the lack of transparency and validation of these company’s tests and results poses a challenge for effective integration into clinical practice. Although the 2019 AHA Scientific Statement initiated the AHA Cardiovascular Genome–Phenome initiative, the guidelines for genomic processing or genetic testing in clinical practice remain poorly defined [124]. Through analyzing genes related to particular heritable conditions and improving prediction models, AI has the potential to facilitate efficient testing of family members and implement precise medicine-based care rather than the current standard practice of diagnosis and treatment based on broad population guidelines.

### 5.2. Variant Calling, Reporting, and Interpretation

Variant calling is used to identify the differences between an individual genome and a reference genome. Despite CLIA approval, there are no guidelines for approval of informatics pipelines for variant calling. There are several variant-related tasks (e.g., read alignment, variant calling, reporting, and interpretation) currently used in genomics screening, the identification of probands, and cascade testing in CVD where AI could be applied. The discrepancies in variant calling between labs, largely because of the lack of clear guidelines, are magnified when undertaking the task of distinguishing true genetic variants from spurious differences introduced by sequencing errors, alignments errors, and other technical artifacts. Other limitations of variant calling include a lack of consensus between variant calling pipelines when analyzing the same data [125], variable accuracies of variant calling algorithms when using different AI technologies, and comparison sequencing of only a limited gene panel. Importantly, AI-driven software, such as DeepVariant, Clairvoyante [38], and Skyhawk [39], have already been used to automatically recognize and prioritize variants with substantially improved accuracy when compared to more traditional statistical models. For example, Google’s DeepVariant uses image recognition techniques and pre-trained models (e.g., inception-v3, variants of CNN model [87]) to pre-process inputs, make inferences, call variants, and then output variant calling format (VCF) files with the variant information. This represents a potential AI solution to the current inconsistencies in variant calling.

Once variants are identified, AI can also help with the interpretation and impact of these variants in clinical practice [126]. For example, SpliceAI [44], DeepBind [127], and DeepSEA [27] can predict the outcomes from different variants with respect to alternative splicing, transcription factor binding, or epigenetic changes, respectively. Additionally, NLP tools have been used in both direct and indirect genetics extraction. For example, BCC-NER [128], and BioNLP [129] have been used for automated extraction of gene and genetic variants or the identification of targeted genes from published literature (Table 2). In CVD specifically, indirect extraction using a family history of sudden cardiac death or HCM using NLP holds promise for better and more efficient management of HCM patients [130]. Most importantly, emerging hybrid models, such as a combination of DL-NLP and deep reinforcement learning, capsule learning, or meta-learning, may overcome the limited knowledge that is currently available to support genomic research. However, a validation of those algorithms is needed first. AI can also be used to collect all clinically relevant information from Medline, the AHA precision medicine platform, or genomic datasets using pre-trained models. However, before that can become reality, a trial of different pre-trained architectures for improved accuracy in variant calling within noisy and imbalanced sequencing data will be needed. 

Variant reporting and interpretation are challenging tasks in clinical cardiovascular practice because, like for variant calling, there are currently few published guidelines in cardiovascular genetics [131]. There are some specific guidelines available, but they only apply to specific genes (e.g., myh7) [132] and are, therefore, not useful in the majority of situations. It is not unreasonable to expect greater guidance in variant interpretation for cardiovascular clinical practice, as other organizations have already released guidelines. For example, the 2015 updated standards and guidelines from the American College of Medical Genetics and Genomics (ACMG) and the Association for Molecular Pathology (AMP) recommended 28 criteria for the clinical interpretation of sequence variants with respect to human diseases. The AHA and ACC should follow this example and develop a statement for genetic testing and a variant interpretation strategy in cardiovascular genetics. 

### 5.3. Combining Genomics with Other Clinical Data Types

Cardiovascular genetics is challenging because both the clinical variables associated with CVDs and the genomics data are heterogeneous and often involve complex interactions between a patient’s genetics and environmental factors. This challenge is largely why applying AI to these multiple types of data is a very promising research direction, and may be especially useful in classifying genome-phenome relationships in CVD using EHRs [133]. For example, combining genomic data describing different septal morphologies of HCM [134,135] with clinical information from echocardiography and angiography could help personalize therapy for individual patients (e.g., deciding if a particular HCM patient needs an ICD). Echo-guided genetic testing or genetic-guided PCI [136] and DAPT duration (e.g., high- vs. low-risk bleeding loci) would also be useful applications of this technology. Another potential application worth researching is the diagnosis of diastolic dysfunction using a combination of echo parameters (e.g., LAVI, E/A ratio, annular e’ velocity, and peak TR velocity) and genetic predispositions since normal diastolic function changes with age [70,71,137]. Precision statin therapy is another potential application for the integration of multiple data types by AI. For instance, in a young female without traditional atherosclerotic risk factors, a combination of genetic testing (e.g., Lp (a), apo C genes) and cardiac imaging (e.g., coronary CT) may reveal a clinical need for preventative statin therapy, which would otherwise never be considered. 

The technical aspects of integrating clinical and genomic data rely on data transformations [138] which convert data into a common vector-matrix format prior to processing using a kernel function. However, this is not the only way to harmonize different data types and modalities. In cardiac amyloidosis, for example, data transformation can be used on echocardiography parameters, immunofixation electrophoresis, and *MAGE CT* genes, and then an ANN can identify the suitability of gene-targeted therapy for patients with equivocal biopsy results. Future research in gene editing therapies for cardiac amyloidosis could be heavily aided and accelerated by AI. In another example, Ross et al. used data transformations to combine 10 SNVs, clinical variables, and laboratory imaging data to predict mortality in peripheral artery diseases using elastic net regression and random forest models [139]. 

AI models can also combine genomic data with data drawn from the EHR and combine them into a unified matrix for clinical analysis. While this strategy is not yet routinely performed, several studies have shown its power and promise. EHR-based phenotyping algorithms have been able to identify familial hypercholesterolemia [140], significant carotid stenosis [141], and the relative prevalence of CAD among different cohorts [142]. Recently, IBM Watson (an automated NLP based algorithm), the Broad Institute of MIT, and Harvard have partnered with the aim of developing AI-based PRS models using population- and hospital-based biobank data, genomic information, and EHRs to identify patients at serious risk for CVD. In addition, ML models have been applied to integrate genetics, cardiac imaging [143], biobank data, and clinical information from EHRs [144] for high-throughput mapping of genotype–phenotype associations to predict diabetes, titin-truncating variants related to DCM [145], and CAD [146]. Another ML study using the Framingham Heart Study cohort used a combination of clinical and genotype data (56 SNPs) for predictive modeling of advanced coronary calcium [75]. By using these examples as a foundation, more advanced studies can be performed with even greater amounts of multidimensional data.

Ultimately, the pipelines of clinical data convergence lie in the ability of AI to unlock multidimensional complex interactions (e.g., gene–environment or gene–behavioral interactions) beyond simply studying gene–gene interactions or host–gut microbiome interactions [147]. For example, air pollutant exposure could lead to changes in DNA methylation and gene silencing without altering the actual DNA sequence [148]. AI could potentially identify relationships between air pollution or zip codes and genes related to detoxification (GSTM1 and GSTT1) or iron processing (HFE), and then generate individualized healthcare recommendations [149]. The combined analysis of these multi-omics data using AI has the potential to provide an improved overall picture of the characteristics of heterogeneous CVDs and, therefore, aid our understanding of their molecular underpinnings. 

### 5.4. Lack of Population Specific Analysis Tools

Across all fields of medicine and research, population-specific analysis tools and databases that can detect population-specific risk factors are urgently needed. Unfortunately, in most cases, including in CV research, significant disparities in research for different ethnicities remain. The pooled cohort equations (PCE) is the cornerstone for atherosclerotic cardiovascular disease (ASCVD) risk stratification and statin treatment decisions [14]. However, the PCE computation mainly focuses on the Caucasian population and overestimates ASCVD risk in Asian and Hispanic populations. Although PCE computations exclude genetic components, the ethnicity disparity is not limited to cardiovascular genetic research [150]. While genomic research in Asian ancestry and African ancestry has increased in recent times [151,152], more than 90% of genomic research has been conducted in patients of mainly European ancestry [153,154]. Furthermore, while most GWAS attempts can control bias of population stratification, fully correcting for population stratification can be challenging and the lack of ethnic diversity included can affect the analysis of gene–environment interactions [155]. Therefore, a major challenge for applying AI more widely is the lack of publicly available non-European genetic databases. In addition, PRS is an emerging technique for assigning genetic risk to individual outcomes that outperforms traditional risk scores [156], but the performance of translating PRS from European ancestry to different ethnicities is largely unknown and not validated [157]. The AI technique of transfer learning could potentially be used to bridge this gap.

A recent study showed that polygenic risk powerfully modifies the risk conferred by monogenic risk variants [158]. However, incorporating these loci into clinical practice is not well established and PRS has limitations in complex disease predictions because of its dependency on linear regression, a lack of phenotype differentiation [159], and a variation in the numbers of SNVs in PRS [160]. A recent quantitative experiment demonstrated some improvement in prediction accuracy using multi-ethnic PRS (mixing training data from Europeans, South Asians, and Africans) [86]. Zhao and Zou investigated PRS, both empirically and theoretically, and found that accuracy can vary dramatically depending on how sparse true genetic signals are [161]. Therefore, an important future research direction is to use AI to explore non-linear PRS relationships, handle interactive high-dimensional data, and randomize selection of SNVs and genetic signals. AI could also be applied to multi-ethnic cohorts to elucidate the role that PRS and ML models, such as GANs, and could potentially play a role in creating a multi-ethnicity PRS. Despite the challenges, some steps have been taken to increase the diversity of WES and WGS samples with efforts such as the Trans-Omics for Precision Medicine (TOPMed) Program, the Million Veteran Program, the Atherosclerosis Risk in Communities (ARIC) Study, the MultiEthnic Study of Atherosclerosis (MESA) [162], and the Multiethnic Variation in Recovery: Role of Gender on Outcomes of Young AMI Patients (VIRGO) Study [163]. Nevertheless, the “unknown unknowns” of modifier genes or polygenic influences in CVDs remains to be explored. Although ML models in the PRS field remain in a developmental phase and have not yet been clinically tested in cardiovascular genetics, AI is poised to overcome current challenges by integrating ethnicity into genomic research.

## 6. Current Limitations in AI Cardiovascular Genetics

Despite steadfast advances, implementing AI in cardiovascular genomics still faces several challenges, including generalizability of results, the required construction of large genomic datasets, and limited computing power. Ultimately, the largest barrier remains the ability of clinicians to implement findings from AI studies.

The first challenge that plagues AI is overfitting an algorithm to a dataset that may adversely affect the generalizability of the results. Generalizability can be partially assessed by evaluating the overfitting of a new dataset. For instance, the results of applying DL models to diabetic retinopathy could not be replicated in different datasets [164,165], and AI methods lack validation data when applied to disease-associated non-coding variants [166,167]. Moreover, many of these mutations have a very small effect on disease risk, even when their combined effects can be clinically relevant. In addition to the ethnic bias discussed previously, AI methods, such as DL, can inadvertently integrate other forms of bias attributed to the training dataset (e.g., bias in word embeddings or variability in extraction algorithms) [168], which represents another challenge in implementing and generalizing results from DL [169,170].

Despite the promise of various AI methods, genomic datasets themselves have built-in limitations: the costs incurred remains a large barrier to performing thorough studies; heterogeneous genetic conditions, such as dilated cardiomyopathy, lack known outputs; and the rarity of specific conditions results in unbalanced case-control studies. These are important limitations when considering the construction of a genomics dataset. Currently, there is not a consensus or indication for genetic testing across several entities within CVD. For patients who undergo genetic testing, the sample can undergo a variety of sequencing techniques that differ between vendors, affecting the quality of the resulting data and confounding interpretation. Moreover, strong evidence of treatment data in cardiovascular genetics is lacking. Premature CAD, for example, with a known or novel actionable mutation may still be treated the same as CAD in older adults by using a high intensity statin, ezetimibe, and/or PCSK9 inhibitor. Identifying a confounder from the CVD-causing relevant environmental factors themselves in genomic data is also challenging, and current DL algorithms have difficulty identifying them as well. Although some DL algorithms can be used in confounder filtering, they cannot be used effectively to control population stratification in GWAS [171,172]. Relatedly, evaluating simulated data or partitioning existing datasets into smaller groups to try and limit confounders may not capture the complexity of genetic data sets and may generate substantially different results.

An equally important barrier to integrating AI study results into clinical practice is the fact that physicians currently lack the necessary access as well as education and training to interpret results from AI studies on genomic data [173,174]. To facilitate clinical adoption, AI can fill the gap in knowledge in clinical practice with automated analysis to detect clinically actionable mutations. However, there is a figurative territorial embargo which limits medical genetics to trained specialists because of the complexity of handling genomic data, rather than a democratization and availability of this technology to all clinicians and patients. Emerging technology, such as homomorphic encryption or blockchains, which can provide an immediate and transparent exchange of encrypted data simultaneously to multiple parties, may be able to fill this gap by at least ensuring data security in handling genomic data. However, there is no process for lifelong interrogation of such data, nor is there specialty infrastructure or funding processes capable of handling that. Most importantly, the main challenge is “trust” in data stewardship. AI has the promise to do automated analyses, but there is no agreement over the format, interpretation, reliability, or reproducibility of the results. 

Despite tremendous recent advances, current quantum or cognitive computing application is still in its infancy. For example, the IBM Watson system has been tasked with identifying and interpreting clinically actionable mutations [175], but still heavily relies on human supervision. Watson’s limitations are likely due to difficulty in integrating with EHRs, too many reported options, and a lack of clinical trials [104,176]. Most importantly, reports from Watson for genomics are based on single-centered studies with weak evidence; they are not based on guidelines and may or may not be beneficial in certain populations or conditions [177]. Notably, the general lack of software infrastructure for genomics-oriented research (e.g., quantum computing, cloud services, supercomputers, or cognitive computing workstations) in cardiology and genetics departments limits the power of AI, even among experts with current access to the data.

Finally, the quality of genomic data between direct-to-consumer companies and clinical or academic institutions may affect the availability and accuracy of “raw data” for AI to analyze. Genotyping data from direct-to-consumer companies, even those that are CLIA certified, contain errors and potentially high false-positive rates (up to 40%) [178]. For example, there is inconsistent labelling of *COL3A1* and *COL5A1* mutations (known to be associated with Ehlers–Danlos syndrome and SCAD) between laboratories [178]. Therefore, standard measures for correlating and combining data from direct-to-consumer and data from clinical or academic institutions are urgently needed. Beyond the technical issues of how variants are reported, there are also substantial privacy concerns involved when sharing genetic data with a direct-to-consumer company. As a minimum, advanced encryption is certainly required to maintain patient privacy. 

## 7. Conclusions

The major barriers to AI-aided genomics reaching widespread clinical practice are fundamentally related to the relative newness of the field itself. Namely, a lack of deep understanding of AI by clinicians, a lack of standardized bioinformatics pipelines, a lack of transparency in AI models, difficulties interpreting the limitations of DL (compared to traditional statistical inferences), problems in structural variations and other complex variant types, unsatisfactory predictive performances in real world genomic problems, a lack of good phenotype data, and poor genomic data quality. In addition, the use of AI-aided genomics research in CVD is also challenged by the heterogeneity of genetic and environmental risk factors. However, with time and further research, these barriers will be overcome, and combinations of AI models will lead to increasingly sophisticated interpretations that may eventually enhance clinical decision making in cardiovascular clinical genetics. Lifestyle data from wearable technology combined with clinical data from EHR and genetic data could tailor treatment towards personalized medicine, ideally identifying CVD at an early stage when it can be more efficiently treated and create a larger improvement in quality of life. In the era of big data, AI-guided studies will translate into increasingly complex genomic datasets, resulting in more sophisticated clinical treatments and improvements in precision medicine. 

## Figures and Tables

**Table 1:** Example of direct-to-consumer genomics companies.

Company	AI algorithms	Input	Database	Limitations	More Information	Example Diseases
23andMe	ML models	Genetic variants	In-house 23andMe database and public databases (e.g., UK Biobank)	Heterogeneity of data (phenotypes, QC control for genetics) between UK Biobank and 23andMe	Map the impact of individuals’ genetic material on phenotypes https://research.23andme.com/publications/ (accessed on 8 February 2022)	Weight pharmacogenetic testing
AncestryDNA	Not specified	Genotype samples on the Illumina OmniExpress platforms	AncestryDNA database	Serious privacy concerns	https://support.ancestry.com/s/article/AncestryDNA-White-Papers (accessed on 8 February 2022)	
Atomwise	ANN model	Gene targets and drug discovery	Public databases and proprietary sources	NA	Predict novel binding compounds; drug discovery ANN model runs an SBVS, which works well with convolution’s ability of extracting local feature clusters from multidimensional input.	Prevent drug related cardiac toxicity
ATUM	ML to develop its Leap-In transposase technology	DNA synthesis Protein Antibody	Protein engineering (ProteinGPS) platform, public domain genetic databases, and proprietary platforms	NA	Enables any recombinant DNA sequence to behave as a transposon (a DNA sequence that can change its position within a genome altering the cell’s genetic identity and genomic size) https://www.atum.bio/resources/archive/presentation-publications (accessed on 8 February 2022)	NA
BenevolentAI	Several models: BioNLP, BERT, deep learning, GuacaMol, Monte Carlo tree search, and symbolic AI		The Reaxys The Chemistry database The ChEMBL database The ZINC database	NA	Understanding the disease mechanisms at the earliest stage of our programs; identify the patients who are likely to respond to a treatment; identify drug targets that control these mechanism(s); and make drugs to correct them https://benevolent.ai/publications (accessed on 8 February 2022)	NA
Calico (Calico Life Sciences LLC)		Proteome Analysis GWAS	AncestryDNA database UK Biobank	NA	www.calicolabs.com/publications/ (accessed on 8 February 2022)	NA
Color Genomics	ML models		Inhouse and industry (e.g., Agilent, Illumina and Hamilton)	No detail of ML model provided	https://www.color.com/wp-content/uploads/2019/12/Color-Hereditary-Heart-Health_WP_v3A.pdf (accessed on 8 February 2022)	Long QT syndrome (LQTS):Left ventricular noncompaction cardiomyopathy Fabry disease
CZ Biohub	ML models	Biochips embedded with human cells	Transcriptome data from animal model	NA	https://www.czbiohub.org/projects/ (accessed on 8 February 2022)	NA
Deep Genomics	Deep Learning	Several types of genetic data	European Genome-Phenome Archive	No detail of DL model provided	Identifying one or more genes responsible for a disease, potential drug therapies for an individual based on genome https://www.deepgenomics.com/platform/ (accessed on 8 February 2022)	Spinal muscular atrophy, nonpolyposis colorectal cancer, and autism
DNAnexus	DeepVariant	NGS data	Public database such as UK Biobank	NA	https://www.dnanexus.com/resources/case-studies (accessed on 8 February 2022)	NA
Fabric Genomics	Proprietary algorithms	NGS	Public database such as gnomAD (gnomad.broadinstitute.org/)	Proprietary model	A proprietary set of algorithms; The Variant Annotation, Analysis and Search Tool (AAST) and Phevor (Phenotype Driven Variant Ontological Re-ranking tool) https://fabricgenomics.com/resources/ (accessed on 8 February 2022)	NA
Freenome	Standard ML models such as logistic regression, principal component analysis (PCA) and support vector machine (SVM)	Whole-genome sequencing, cfDNA, cfRNA, and protein data	Proprietary sources and public database (e.g., NIH Roadmap Epigenome Mapping Consortium)	Proprietary sources	AI-EMERGE (NCT03688906)	NA
Futura Genetics		DNA from saliva		NA	APEX (arrayed primer extension) technology for detecting SNPs	NA
Genoox	AI-based variant classification (aiVCE)	NGS	In-house exome database; public and in-house variant databases	NA	Diagnosis and treatment of genetic disorders and cancer, as well as new drug discovery and family planning; automated classification engine based on ACMG guidelines https://www.genoox.com/publications/ (accessed on 8 February 2022)	NA
Grail				NA	The Circulating Cell-free Genome Atlas (CCGA) Study The STRIVE Study SUMMIT Study https://grail.com/science/publications/ (accessed on 8 February 2022)	NA
IBM Watson for Genomics	NLP for several different predictive models	VCFs, CNV, and gene expression data abstracts and full-text articles	In-house hospital, PubMed and ClinicalTrials.gov	NA	Driver alterations, actionable variants, VUS, relevant therapies, and potential clinical trials https://www.ibm.com/us-en/marketplace/watson-for-genomics (accessed on 8 February 2022)	glioblastoma
Illumina	SpliceAI PrimateAI: deep residual neural network	NGS	Public databases (e.g., the ExAC/gnomAD database; the Single-Nucleotide Polymorphism Database (dbSNP); and ClinVar database	NA	Distinguish a handful of disease-causing mutations in patients with rare genetic diseases from a large number of benign variants present in healthy people https://www.illumina.com/science/publication-reviews.html (accessed on 8 February 2022)	NA
Karius	Proprietary Karius AI technology	blood test based on next-generation sequencing	NA	Proprietary model	https://www.kariusdx.com/clinical-data#publications (accessed on 8 February 2022)	endocarditis
Nvidia and Scripps Research Translational Institute	Deep Learning	Development phase	NA	Still in development phase and not many details disclosed	Blood pressure monitoring; blood glucose genomics; digital wearable data	NA
Quest Diagnostics	Watson’s cognitive computing and hc1’s machine learning technology	Genome sequencing	In-house	No detail of ML model provided	https://www.hc1.com/blog/tag/quest-diagnostics/ (accessed on 8 February 2022)	NA
SOPHiA Genetics	Proprietary and standard algorithms (e.g., hidden Markov model algorithm)	NGS data	In-house and public databases (e.g., ClinVar, ExAC, and dbSNP)	NA	SNVs, Indels and CNVs detection, ALU insertions, Pseudogene variants differentiation and variant annotation https://www.sophiagenetics.com/en_US/hospitals/solutions/solutions/CAS.html (accessed on 8 February 2022)	arrhythmias (e.g., Long/Short QT syndrome or Brugada syndrome) and cardiomyopathies
Synpromics	ML models	Gene promoter design, a novel genomics-based platform	BIOBASE Biological Databases, UCSC GoldenPath, European Bioinformatics Institute	No detail of ML model provided	Predict the genomic sequences that are involved in cell type-specific regulation of gene expression	Design of Synthetic Mammalian Promoters
Verge Genomics	AI in pharmacogenomics	microRNA (miRNA)	Academic databases, research centers, and public databases (e.g., the NCBI database and the Molecular Signatures Database (MSigDB))	Proprietary AI model	AI-generated therapies for ALS and Parkinson by screening thousands genes https://www.vergegenomics.com/publications (accessed on 8 February 2022)	NA
Verily	DeepMass Project Baseline Health Study Status	Protein signals, genomics, and transcriptomics	Identify and quantify proteins	No validation	Integrate protein signals with other biomolecular data, such as genomics and transcriptomics, as well as with device measurements and disease status, to find out how genetics and behavior affect protein profiles https://blog.verily.com/2019/05/deepmass-new-machine-learning-method.html (accessed on 8 February 2022)	NA
Veritas Genetics	ML models and AI Arvados Data Platform	Whole Genome Sequencing and Whole Exome Sequencing	Internal databases of two clinical testing laboratories (Laboratory for Molecular Medicine and Veritas Genetics) and public databases (e.g., ClinVar)	NA	https://www.veritasgenetics.com/in-the-news (accessed on 8 February 2022)	NA
Viome	Watson machine-learning	Gut microbiome	NA	No publications seen in Pubmed	https://www.viome.com/our-science (accessed on 8 February 2022)	NA

**Table 2:** Examples of variant calling, reporting, and interpretation AI.

Name	Algorithms	Example Function
DeepVariant [37]	Deep convolutional neural network (CNN)	Variant calling from short-read sequencing by reconstructing DNA alignments as an image
Clairvoyante [38]	A multi-task convolutional deep neural network	(1) Variant calling in single molecule sequencing (2) Predicts variant types (SNP or indel), zygosity, and alleles at the same time
Skyhawk [39]	Neural network	Mimics the process of expert review for clinically significant genomics variants identification
DeepBind [40]	Deep CNN	Predicts the binding sites of DNA-binding proteins and RBPs
iDeep [41]	Deep belief networks (DBN) and CNN	Cross-domain features and sequence information
DeepSEA [42]	Deep CNN	Predicts functional consequences of noncoding variants
DeepNano [43]	Recurrent neural networks (RNN)	Base calling in MinION nanopore reads
SpliceAI [44]	Deep neural network (DNN)	(1) Predicts splice junctions from an arbitrary pre-mRNA transcript sequence (2) Predicts noncoding genetic variants that cause cryptic splicing
DeepGestalt [45]	DNN	Distinguishes more than 200 rare diseases based on patient face images, which could also separate different genetic subtypes (e.g., Noonan syndrome)
DeepPVP [46]	DNN	Variant prioritization by integrating patients’ phenotype information
DeepSVR [47]	Deep learning and random forest models	Predicts somatic variants confirmed by orthogonal validation sequencing data
DeepGene [48]	DNN	Extracts the high-level features between combinatorial somatic point mutations and cancer types. Classify cancer type
Deep AE [49]	Autoencoder	gene expression data
DeepMethyl [50]		Predicts methylation states of DNA CpG dinucleotides
BioVec [51]		Feature representation
DeepMotif [52]	Deep convolutional/highway MLP framework	Sequential data about gene regulation
DeepChrome [53]	Deep CNN	Sequential data about gene regulation Classifies gene expression using histone modification data as input.
Chiron [54]	Deep learning model	Translates the raw signal to DNA sequence
Variational Autoencoders [55]	Autoencoder	Predicts drug response
GARFIELD-NGS [56]	Deep CNN	Dissects false and true variants in exome sequencing
DeepGS [57]	Deep CNN	Predicts phenotypes from genotypes
DANN [58]	DNN	Predicts deleterious annotation or pathogenicity of genetic variants
DanQ [59]	Hybrid model Deep RNN and CNN	Quantifies the function of non-coding DNA
ProLanGO [60]	RNN	Protein function prediction
BCC-NER [61]	NLP	Bidirectional and contextual clues named entity tagger for gene/protein mention recognition
BioNLP [62]	NLP	Gene regulation network
SpaCy [63]	NLP	Tagging, parsing, and entity recognition

## Abbreviations

ARVC	arrhythmogenic right ventricular cardiomyopathy
AI	artificial intelligence
ANN	artificial neural network
CVD	cardiovascular disease
CLIA	Clinical Laboratory Improvement Amendment
CAP	College of American Pathologists
CNN	convolutional neural network
CNV	copy number variation
CAD	coronary artery disease
DL	deep learning
DCM	dilated cardiomyopathy
DAPT	dual antiplatelet therapy
DNN	deep neural network
EHR	electronic health record
FDA	Food and Drug Administration
GAN	generative adversarial network
GWAS	genome-wide association studies
HFmrEF	heart failure with midrange ejection fraction
HFpEF	heart failure with preserved ejection fraction
HFrEF	heart failure with reduced ejection fraction
HCM	hypertrophic cardiomyopathy
ICD	implantable cardioverter defibrillator
LAVI	left atrial volume index
ML	machine learning
NLP	natural language processing
NGS	next generation sequencing
PCI	percutaneous coronary intervention
PRS	polygenic risk score
QC	quality control
RNN	recurrent neural network
SNP	single nucleotide polymorphisms
SNV	single nucleotide variant
SCAD	spontaneous coronary artery dissection
TR	tricuspid regurgitation
UK	United Kingdom
USA	United States of America
VCF	Variant Calling Format
WES	whole exome sequencing
WGS	whole genome sequencing
